# COVID-19 in cancer patients: clinical characteristics and outcome—an analysis of the LEOSS registry

**DOI:** 10.1007/s00277-020-04328-4

**Published:** 2020-11-07

**Authors:** Maria Madeleine Rüthrich, C. Giessen-Jung, S. Borgmann, A. Y. Classen, S. Dolff, B. Grüner, F. Hanses, N. Isberner, P. Köhler, J. Lanznaster, U. Merle, S. Nadalin, C. Piepel, J. Schneider, M. Schons, R. Strauss, L. Tometten, J. J. Vehreschild, M. von Lilienfeld-Toal, G. Beutel, K. Wille, on behalf of the LEOSS Study Group

**Affiliations:** 1 Department of Internal Medicine II, Hematology and Medical Oncology, University Hospital Jena, Am Klinikum 1, 07747 Jena, Germany; 2 Leibniz Institute for Natural Product Research and Infection Biology, Hans-Knöll Institute, Jena, Germany; 3 Department of Internal Medicine III, Ludwig Maximilian University, Munich, Germany; 4 Department of Infectious Diseases and Infection Control, Ingolstadt Hospital, Ingolstadt, Germany; 5 Department I for Internal Medicine, University of Cologne, Faculty of Medicine and University Hospital Cologne, Cologne, Germany; 6 German Centre for Infection Research (DZIF), partner site Bonn-Cologne, Cologne, Germany; 7 Department of Infectious Diseases, University Hospital Essen, University Duisburg Essen, Essen, Germany; 8 Section Clinical Infectiology, University Hospital Ulm, Ulm, Germany; 9 Emergency Department, University Hospital Regensburg, Regensburg, Germany; 10 Department of Internal Medicine II, Division of Infectious Diseases, University Hospital Würzburg, Würzburg, Germany; 11 Cologne Excellence Cluster on Cellular Stress Responses in Aging-Associated Diseases (CECAD), University of Cologne, Cologne, Germany; 12 Department of Internal Medicine II, Passau Hospital, Passau, Germany; 13 Department of Internal Medicine IV, University Hospital Heidelberg, Heidelberg, Germany; 14 Department of General, Visceral and Transplant Surgery, University Hospital Tübingen, Tübingen, Germany; 15 Hospital Bremen-Center, Bremen, Germany; 16 Department of Internal Medicine II, Technical University of Munich, School of Medicine, University hospital rechts der Isar, Munich, Germany; 17 Cologne Excellence Cluster on Cellular Stress Responses in Aging-Associated Diseases (CECAD), University of Cologne, Cologne, Germany; 18 Medical Clinic I, University Hospital Erlangen, Erlangen, Germany; 19 Department of Gastroenterology and Infectiology, Hospital Ernst-von-Bergmann, Potsdam, Germany; 20 Department of Internal Medicine, Hematology/Oncology, Goethe University Frankfurt, Frankfurt am Main, Germany; 21 Department of Hematology, Hemostasis, Oncology and Stem Cell Transplantation, Hannover Medical School, Hannover, Germany; 22 University of Bochum, University Clinic for Hematology, Oncology, Hemostaseology and Palliative Care, Minden, Germany

**Keywords:** COVID-19, SARS-CoV-2, Cancer patients, Pandemic, LEOSS, Cohort study, Adjusted analysis

## Abstract

**Introduction:**

Since the early SARS-CoV-2 pandemic, cancer patients have been assumed to be at higher risk for severe COVID-19. Here, we present an analysis of cancer patients from the LEOSS (Lean European Open Survey on SARS-CoV-2 Infected Patients) registry to determine whether cancer patients are at higher risk.

**Patients and methods:**

We retrospectively analyzed a cohort of 435 cancer patients and 2636 non-cancer patients with confirmed SARS-CoV-2 infection, enrolled between March 16 and August 31, 2020. Data on socio-demographics, comorbidities, cancer-related features and infection course were collected. Age-, sex- and comorbidity-adjusted analysis was performed. Primary endpoint was COVID-19-related mortality.

**Results:**

In total, 435 cancer patients were included in our analysis. Commonest age category was 76–85 years (36.5%), and 40.5% were female. Solid tumors were seen in 59% and lymphoma and leukemia in 17.5% and 11% of patients. Of these, 54% had an active malignancy, and 22% had recently received anti-cancer treatments. At detection of SARS-CoV-2, the majority (62.5%) presented with mild symptoms. Progression to severe COVID-19 was seen in 55% and ICU admission in 27.5%. COVID-19-related mortality rate was 22.5%. Male sex, advanced age, and active malignancy were associated with higher death rates. Comparing cancer and non-cancer patients, age distribution and comorbidity differed significantly, as did mortality (14% vs 22.5%, *p* value < 0.001). After adjustments for other risk factors, mortality was comparable.

**Conclusion:**

Comparing cancer and non-cancer patients, outcome of COVID-19 was comparable after adjusting for age, sex, and comorbidity. However, our results emphasize that cancer patients as a group are at higher risk due to advanced age and pre-existing conditions.

**Supplementary Information:**

The online version contains supplementary material available at 10.1007/s00277-020-04328-4.

## Introduction

Since the emergence of severe acute respiratory syndrome coronavirus 2 (SARS-CoV-2) in December 2019 in Wuhan, global number of coronavirus disease 2019 (COVID-19) cases are steadily increasing [1]. As of October 12, 2020, approximately 4.1 million cases and 195,650 deaths related to COVID-19 have been reported in the EU/EEA and the UK [2].

In COVID-19, clinical findings can range from mild flu-like symptoms to life-threatening respiratory insufficiency. Average incubation period is 5–6 days, while the period between infection onset to hospitalization is reported as 7, to respiratory deterioration 8, and to ICU admission 10 days [3, 4]. Although cancer and non-cancer patients appear to have similar infection rates, it is generally assumed that cancer patients are at higher risk for severe COVID-19 and death attributed to COVID-19 [5–8]. Numerous studies identified advanced age, male sex, and a high number of comorbidities as risk factors for a worse outcome [6, 9–11]. Additionally, cancer-related features, such as immunodeficiency due to the underlying malignancy, effects of anti-cancer therapies, and frequent contacts with healthcare workers are discussed being associated with higher mortality and severe infection course [9, 12, 13].

Although detailed descriptions and analyses of risk factors, clinical courses, and mortality in cancer patients infected with SARS-CoV-2 exist, published literature is heterogeneous regarding tumor entities, anti-cancer treatment and matched confounders [5, 9, 12–15]. Moreover, comparisons of cancer and non-cancer patients are scarce.

Here, we present an analysis of SARS-CoV-2-infected cancer patients from the Lean European Open Survey on SARS-CoV-2 (LEOSS) registry. We aimed to describe epidemiological and clinical features and to determine whether cancer patients are at higher risk for severe infection course and COVID-19 mortality compared to non-cancer patients.

## Patients and methods

In March 2020, the multicenter Lean European Open Survey on SARS-CoV-2 infected patients (LEOSS) registry was established in order to quickly gain knowledge about the epidemiology and clinical course of patients infected with SARS-CoV-2.

We retrospectively assessed the data of cancer patients and a non-cancer control cohort with (PCR) confirmed SARS-CoV-2 infection, who were hospitalized or treated in an outpatient setting at a LEOSS partner site. From a total of 3071 patients, enrolled between March 16 and August 31 2020, 435 cancer patients could be identified. Only patients with available data on follow-up or last known status were included. Baseline patients’ data were collected on socio-demographics, the presence of comorbidity according to Charlson Comorbidity Index (CCI), and cancer-related features, including entity, Eastern Cooperative Oncology Group Index (ECOG), disease status at detection of SARS-CoV-2 (active disease, chemotherapy, high-dose steroids, targeted therapy, other immunosuppressive medication, remission) and information about the anti-cancer therapy. Unfortunately, information on causative treatment of COVID-19 from this early phase of the pandemic was not available in a systematic and controlled fashion. We therefore did not include any analysis on putative therapeutic effects.

Clinical manifestation of COVID-19 was described in four phases: uncomplicated (oligo-/asymptomatic), complicated (need for oxygen supplementation), critical (need for life supporting therapy), and recovery (clinical improvement/discharge, Table 1). Symptoms, vital signs, and laboratory values were analyzed over all phases as was outcome of SARS-CoV-2 infection. Primary endpoint was death attributed to COVID-19. Secondary endpoints were survival at day 30 and outcome from SARS-CoV-2 infection after adjustments for age, sex, and comorbidity.

**Table 1:** LEOSS clinical phases

Uncomplicated phase • Asymptomatic OR • Symptoms of upper respiratory tract infection • Gastrointestinal symptoms • Fever	Recovery phase • Improvement by one degree of severity according scheme of LEOSS clinical phases or discharge AND • Defervescence AND • No progression/re-hospitalization
Complicated phase	Critical phase
• Need for oxygen supplementation	• Need for catecholamines
• Increase of prior oxygen home therapy	• Life-threatening cardiac arrhythmia
• paO2 < 70 mmHg/SO2 < 90% (at room air)	• Need for unplanned mechanical ventilation
• Transaminases > 5× ULN	• Prolongation of planned mechanical ventilation
• New cardiac arrhythmia	• Liver failure (Quick < 50%)
• New pericardial effusion (> 1 cm)	• qSOFA ≥ 2
• New heart failure with pulmonary edema, congestive hepatopathy, peripheral edema	• Acute renal failure in need of dialysis

### Data collection and statistical analysis

Anonymized data was collected in an electronic case report form (eCRF) using the online platform ClinicalSurveys.net, which was developed by the University Hospital of Cologne (UHC). Data management and statistical analysis was performed with SPSS software version 27.0 (IBM, Corp. in Armonk, NY). Categorical variables were expressed using counts and percentages. Continuous data were presented as mean with standard deviation (±SD) or as median with interquartile range (IQR). To compare categorical variables, Pearson’s chi-square test was used. Two-sided *t* test was used to compare continuous data. Survival was estimated using the Kaplan-Meier method (log-rank test). Patients were adjusted for age, sex, and comorbidity. Significance level was set at 0.05.

## Results

### Baseline characteristics

A total of 435 cancer patients with SARS-CoV-2 infection treated at a LEOSS study site were included in our analysis. The majority of patients were hospitalized (98%, 427/435). Detailed patients’ characteristics are shown in Table 2. Median observational period was 14 days (IQR 7 - 24) and median duration of hospitalization 15 days (IQR 8 – 27.5). More men than women were included (59.5% vs 40.5%). Most common age category was 76–85 (36.5%). A male predominance was seen across all age categories except for patients > 85 years (female 19/38 vs male 19/38). In 23.5% (44/187), ECOG was > 2. Mean CCI was 4.64, while common comorbidities taken into account were chronic kidney disease and cardiovascular diseases. Arterial hypertension was seen in 60%. Solid tumors were documented for 256 (59%) and lymphoma and leukemia for 76 (17.5%) and 48 (11%) patients, respectively. Common solid tumor entities were gastrointestinal cancer (14%, 60/421), lung cancer (8.5%, 36/421), gynecological and breast cancer (9%, 39/421 and 9.5%, 21/421, respectively). Metastasized solid tumors were seen in 95 (22%, 95/435) patients. Commonest hematological malignancies were non-Hodgkin lymphoma (16.5%, 71/421) and acute myeloid leukemia (3%, 14/421). About half of patients had an active malignancy at detection of SARS-CoV-2, one-quarter had received anti-cancer treatments within the last 3 months. Type of therapy was almost equally distributed between chemotherapy, radiation, and surgery.

**Table 2:** Patients’ characteristics

	Cancer patients, *N* = 435	Non-cancer patients, *N* = 2636	*p* value
Age categories; n/N (%)			< 0.001
< 18 years	5/435 (1)	47/2636 (2)	
18–25 years	1/435 (0.5)	60/2636 (2)	
26 – 35 years	4/435 (1)	197/2636 (7.5)	
36 – 45 years	13/435 (3)	239/2636 (9)	
46 – 55 years	36/435 (8)	434/2636 (16.5)	
56 – 65 years	72/435 (16.5)	527/2636 (20)	
66 – 75 years	108/435 (25)	413/2636 (15.5)	
76 – 85 years	158/435 (36.5)	508/2636 (19.5)	
> 85 years	38/435 (8.5)	211/2636 (8)	
Sex; n/N (%)			0.952
Female	176/435 (40.5)	1073 /2636 (40.5)	
Male	259/435 (59.5)	1563/2636 (59.5)	
CCI (±SD)			
CCI mean	4.65 (±2.68)	1.12 (±1.77)	< 0.001
CCI w/o cancer	1.59 (±1.99)	1.12 (±1.77)	< 0.001
Comorbidities; n/N (%)			
Hemiplegia	15/431 (3.5)	55/2562 (2)	0.090
Dementia	48/429 (11)	209/2358 (8)	0.037
Cerebrovascular disease, Stroke, TIA	59/427 (14)	222/2560 (8.5)	0.001
Myocardial infarction	36/424 (8.5)	156/2539 (6)	0.069
Chronic heart failure	52/420 (12)	242/2537 (9.5)	0.071
Peripheral vascular disease	34/421 (8)	104/2521 (4)	< 0.001
Hypertension	259/430 (60)	1249/2591 (48)	< 0.001
Coronary artery disease	81/420 (19.5)	343/2518 (13.5)	0.002
COPD	39/430 (9)	149/2572 (6)	0.009
Asthma	15/429 (3.5)	137/2564 (5.5)	0.107
Other chronic pulmonary disease	27/422 (6.5)	110/2539 (4.5)	0.061
Connective tissue disease	3/429 (0.5)	20/2560 (1)	0.857
Peptic ulcer disease	19/428 (4.5)	51/2558 (2)	0.002
Chronic liver disease	5/428 (1)	53/2558 (2)	0.210
Liver cirrhosis	6/428 (1.5)	22/2561 (1)	0.281
Diabetes w/o end organ damage	63/429 (14.5)	349/2572 (13.5)	0.534
Diabetes w end organ damage	41/426 (9.5)	172/2563 (6.5)	0.030
Chronic kidney disease	87/428 (20.5)	359/2569 (14)	0.001
Acute kidney injury	25/328 (7.5)	121/1878 (6.5)	0.428
Organ transplantation	5/429 (1)	49/2564 (2)	0.283
Obesity; n/N (%)			
BMI > 30 kg/m^2^	69/184 (24)	419/1597 (26)	0.453
Smoking; n/N (%)			0.044
Active smoking	36/225 (16)	170/1270 (14)	
Non-smoking	141/225 (62.5)	899/1270 (70)	
Former smoking	48/225 (21.5)	201/1270 (16)	
Poor performance status			
ECOG > 2; n/N (%)	44/187 (23.5)		
Underlying cancer disease (according to CCI); n/N (%)			
Solid tumor	256/435 (59)		
Solid tumor, metastasized	95/435 (22)		
Lymphoma	76/435 (17.5)		
Leukemia	48/435 (11)		
Disease status at detection of SARS-CoV-2; n/N (%)			
Active disease	193/359 (54)		
Chemotherapy	71/357 (20)		
High-dose steroids	27/340 (8)		
Targeted therapy	48/341 (14)		
Other immunosuppressive therapy	36/343 (10.5)		
Remission	59/117 (50.5)		
Anti-cancer therapy (**≤** 1 month)	96/418 (22)		
Chemotherapy (**≤** 3 months)	36 /418(8.5)		
Surgery (**≤** 3 months)	39/418 (9)		
Radiation (**≤** 3 months)	25/418 (5.5)		

*CCI* Charlson Comorbidity Index, *w/o* without, *BMI* body mass index, *ECOG* Eastern Cooperative Oncology Group

### Clinical course of COVID-19

At the time of a positive test for SARS-CoV-2, 272 (63%) patients were in an uncomplicated phase (according to LEOSS clinical stages, suppl. Table 1). Most common symptoms were fever (34%, 148/398), dry cough (24.5%, 106/398), dyspnea (23.9%, 104/379), and excessive tiredness (18.9%, 82/398). Fifty-eight (13.5%, 58/398) patients were reported to be asymptomatic. Signs of advanced stages were described in a minority of patients: oxygen saturation < 90% in 15.5%, systolic blood pressure < 80 mmHg in 0.5%, diastolic blood pressure < 60 mmHg in 12.5%, and heart rate ≥ 90 bpm in 35.5% of the patients. Anemia was reported in 56% and lymphopenia and neutropenia in 83.5% and 18%, respectively. C-reactive protein (CRP) was above 120 mg/L in 66 (18.5%) patients.

Progression to complicated or critical phases were observed in 206 (55%, 206/376) patients. One-hundred and nineteen (27.5%, 119/435) patients were admitted to ICU with 78 (65.5%, 78/119) patients receiving mechanical ventilation. Median ICU length of stay was 11 days (IQR 7.75 – 27). Along with progression to the critical phase, oxygen saturation decreased below 90% in 79%, while systolic blood pressure less than 80 mmHg and diastolic blood pressure less than 60 mmHg were found in 30.5% and 74%, respectively. In 73.5%, tachycardia was observed. In patients with available laboratory values, 69% had CRP levels above 120 mg/L, while leukopenia was found in one-fifth (21%) and lymphopenia and neutropenia in 89% and 20%, respectively. Half of patients had a severe anemia (< 8 g/dL, 51%). Disease progression to complicated or critical phases was not associated with an active cancer disease at detection of SARS-CoV-2 (*p* values 0.194 and 0.718, respectively).

**Fig. 1:**
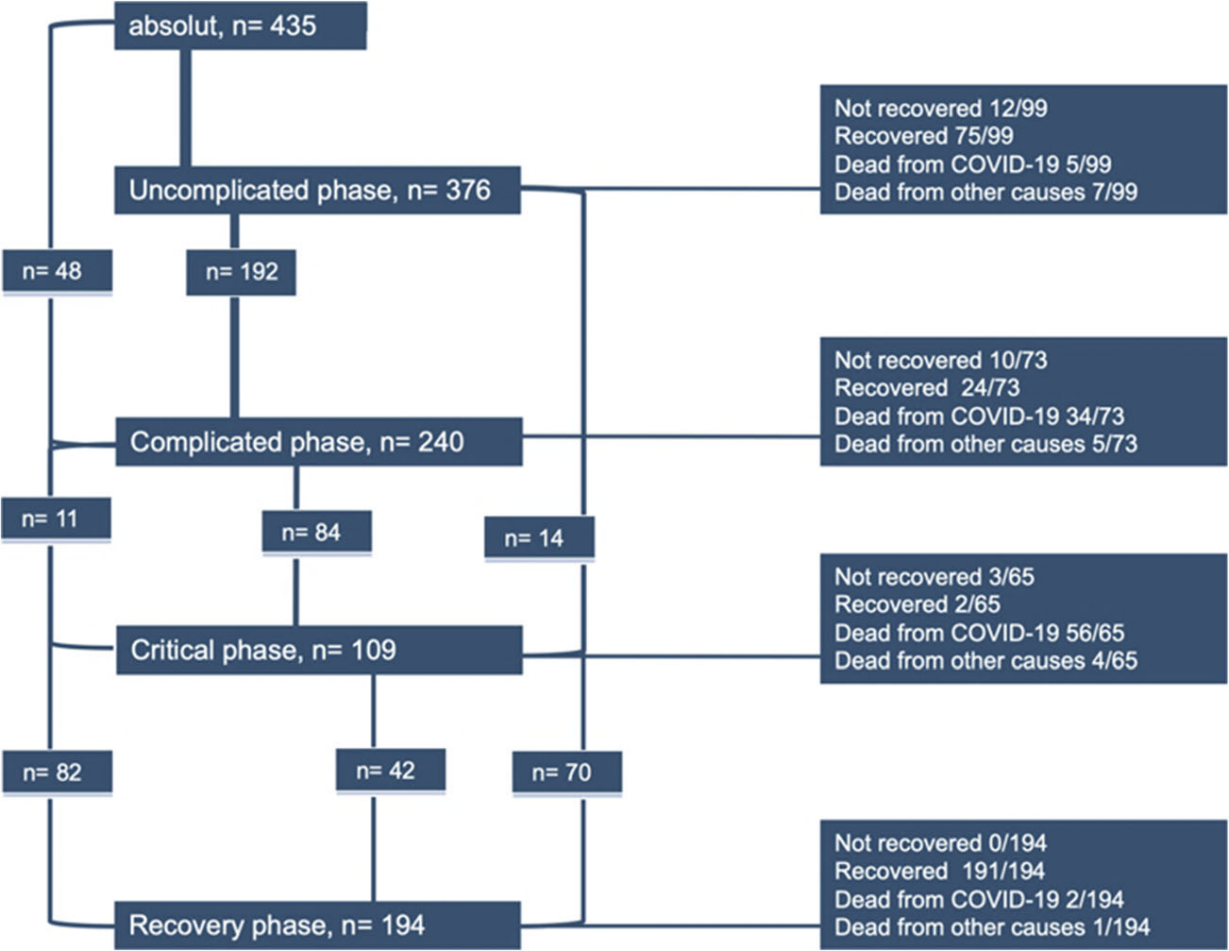
Phases of COVID-19

Of all patients, 194 (44.5%) patients reached the recovery phase, whereas clinical improvement or discharge was reported for a total of 292 (67.5%) patients (Fig. 1).

### Survival analysis

**Fig. 2:**
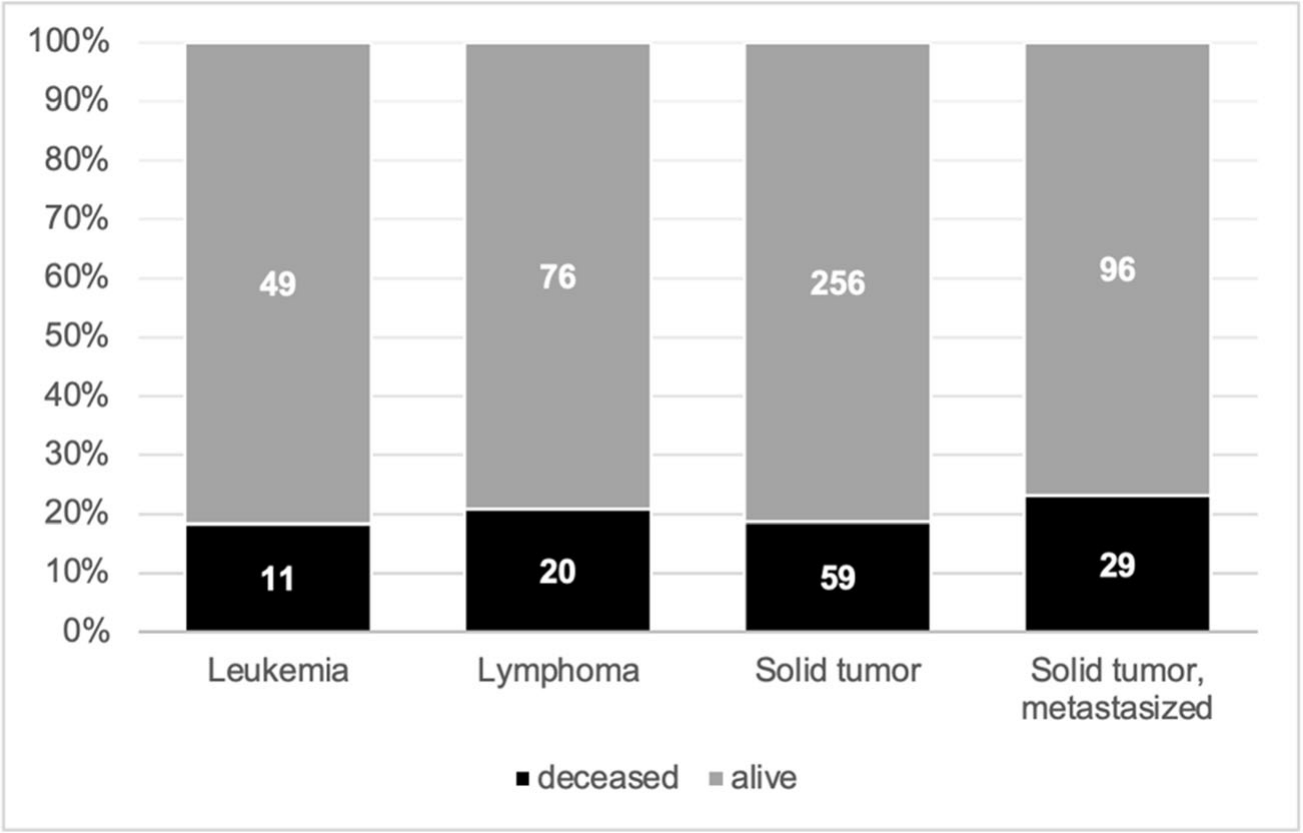
Mortality rate attributed to COVID-19

Kaplan-Meier estimates showed a median survival of 48 days (95% CI 33.5–62.4) for all cancer patients and 31 days (95% CI 22.6–39.3) for ICU patients. The primary endpoint (death related to COVID-19) was observed in 97 (22.5%) patients, while the majority died within 30 days after diagnosis of SARS-CoV-2 (19.5%). The mortality rate was significantly higher in male patients (28%, 73/216 vs 13.5%, 24/177; *p* value < 0.001) and in patients with an active cancer disease at detection of SARS-CoV-2 (26.5%, 52/197 vs 17%, 28/167; *p* value 0.027). In solid tumors and hematological malignancies, mortality was comparable (Fig. 2). The highest death rates were seen in patients aged 76–85 (31.5%, 50/158) and in patients > 85 years (37%, 14/38). In the age categories 56–65 years and < 55, COVID-19-related mortality was 11% (8/72) and 9% (5/55), respectively.

In the group of 119 patients treated on an ICU, 47 (39.5%) patients died attributed to COVID-19, 37 (31%) within 30 days after detection of SARS-CoV-2. Again, mortality was higher in males (57.5%, 23/40 vs 33.5%, 5/15; *p* value 0.103) and slightly higher in patients with an active cancer disease (50%, 13/26 vs 43%, 9/21; *p* value 0.626). The majority of patients who died was aged 66-85 (74.5%, 35/47). Although 28/38 patients > 85 years showed progression to severe COVID-19 phases, ICU admission was seen only in one.

Of all patients, who died attributed to COVID-19, 15 (18%, 15/83) patients were on palliative care before positively tested for SARS-CoV-2. In 76.5% (66/86), treatment goals were changed from curative to palliative, mainly due to progression of COVID-19. Both were predominantly observed in patients aged 76 and older.

**Fig. 3:**
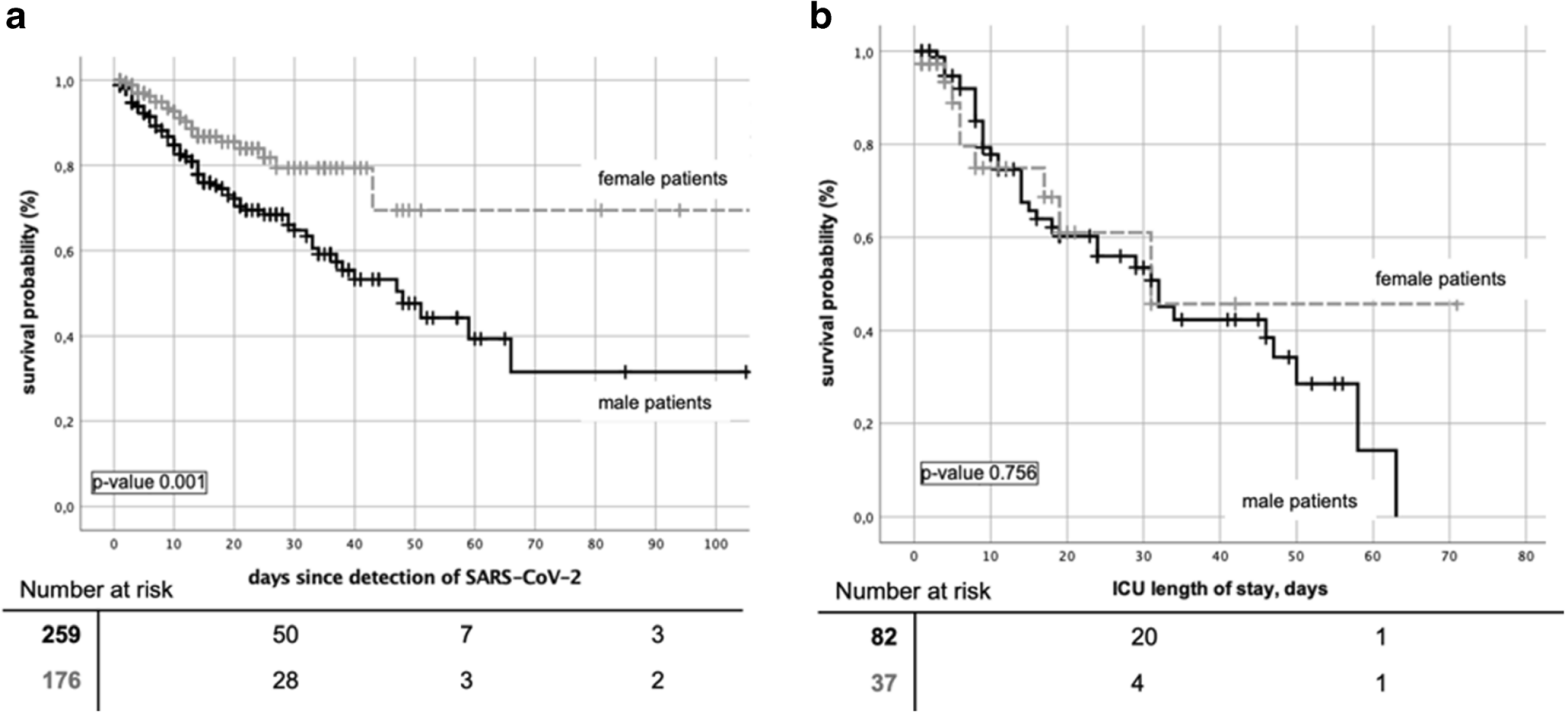
Kaplan-Meier survival comparing female vs male in (**a**) all cancer patients and (**b**) cancer patients on intensive care units

In univariate analysis, male sex (Fig. 3) and advanced age (*p* value 0.005) were predictive for a worse outcome, whereas an active disease or high-dose steroids at detection of SARS-CoV-2 did not influence survival significantly (*p* values 0.060 and 0.907, respectively).

**Fig. 4:**
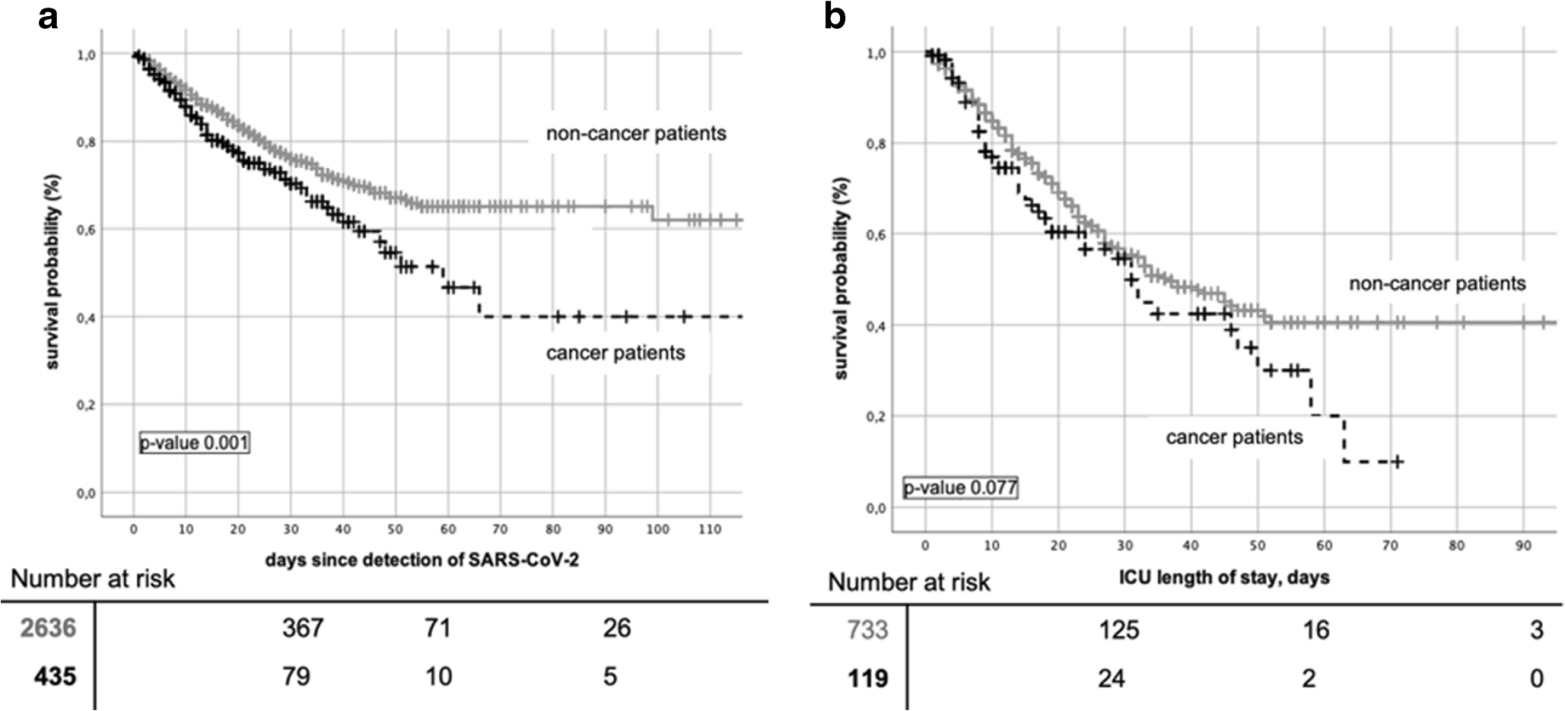
Kaplan-Meier curves of overall survival comparing cancer vs non-cancer patients in (**a**) all patients and (**b**) patients on intensive care units

**Fig. 5:**
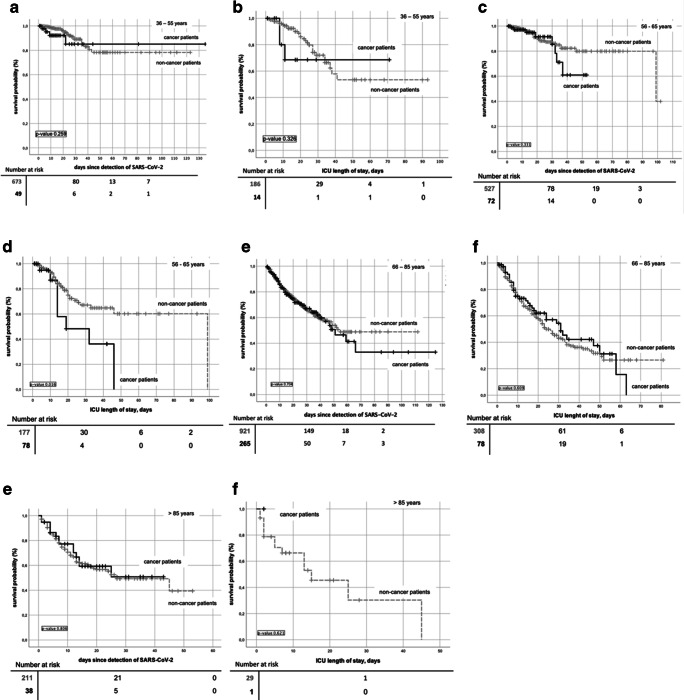
Kaplan-Meier curves of overall survival for all patients and patients on ICU in (**a**–**b**) patients aged 35–55, (**c**–**d**) patients aged 56–65 patients, (**e**–**f**) patients aged 66–85, and (**g–h**) patients > 85 years of age

In order to compare clinical courses of cancer and non-cancer patients, the group of cancer patients was compared to a total of 2636 non-cancer patients included into LEOSS in the same time period. Data of non-cancer patients are shown in Table 2. More men than women were included (59.5% vs 40.5%); most common age category was 56–65 (20%). Mean CCI was 1.1. Comparing survival of both groups, cancer patients showed a significantly worse outcome (Fig. 4). Thus, survival at day 30 was 77% (95% CI 74.35–79.45) and 70.5% (95% CI 64.2–76.4, *p* value 0.001) in non-cancer and cancer patients, respectively. In patients on intensive care treatment, Kaplan-Meier estimates showed a 30-day-survival of 56.5% (95% CI 51.5–62) in non-cancer and 54.5 (95% CI 42.9–60.1, *p* value 0.077) in cancer patients. Mortality attributed to COVID-19 was significantly higher in cancer patients (14%, 367/2636 vs 22.5%, 97/435; *p* value < 0.001). Comparing baseline data of both groups, age distribution and prevalence of comorbidity differed significantly (*p* value < 0.001 each, Table 2) as non-cancer patients were younger and had less comorbidities. To account for those differences, survival analysis was adjusted for age, sex, and comorbidity. Four groups with almost equally distributed confounders were identified (suppl. Table 2). Across these groups, no significant differences in survival were seen except for ICU patients aged 56–65. Here, 30-day survival was 67% (95% CI 57.1–77.1) in non-cancer patients and 48% (95% CI 18.4–78, *p* value 0.016, Fig. 5) in cancer patients, respectively. Mortality rates were comparable, while highest death rates were seen in ICU patients greater than 65 and in patients greater than 85 years of age.

## Discussion

This multicenter cohort study provides detailed information on epidemiological and clinical characteristics of cancer patients compared to non-cancer patients, both positively tested for SARS-CoV-2. Our cohort consisted of 435 cancer and 2636 non-cancer patients, respectively. Patients of both groups required medical care at a LEOSS study site.

LEOSS is a multicenter cohort study, which is characterized by an open and collaborative approach. By now, LEOSS registers 256 active European and non-European study sites [16]. Highest documentation rates were performed by universities and tertiary care hospitals in larger cities. As a consequence, rural areas and community practices are underrepresented causing a bias towards severe cases. Over the course of the COVID-19 pandemic, medical knowledge steadily grows. Therefore, eCRFs are regularly updated and extended. As a result, data of patients documented in the early pandemic are incomplete, and absolute numbers vary.

Since most participating sites were located in Germany, only a few patients from other countries were included, and generalizability of our results might be limited [16].

Socio-demographics and comorbidity of our cancer patients were well comparable with previously published literature. Thus, our cohort was characterized by patients aged 65 years and older and a male predominance [5, 9, 12, 13, 17–19]. Commonest comorbidity was hypertension, followed by chronic kidney disease and diabetes [5, 9, 12, 14].

As reported in other studies, half of our patients had an active cancer disease, while one out of every 20 patients had received an anti-cancer treatment within 1 month before detection of SARS-CoV-2 [13, 14].

Even though solid tumors were more frequent, hematological malignancies were overrepresented compared to a non-COVID-19 cancer cohort from the UK reporting a prevalence of 9.5% [19]. It can thus be reasonably assumed that patients with hematological malignancies are more often exposed to the healthcare environment, where thresholds for testing are lower in hospital settings. Additionally, Lee at al. reported an increased susceptibility to viral infections in patients with hematological malignancies [19].

At detection of SARS-CoV-2, most patients were in an uncomplicated phase, which was in accordance to earlier reported patient cohorts, including cancer and non-cancer patients [20, 21]. Similar to published data, anemia, lymphopenia, and elevated CRP were common findings in cancer patients [13, 17].

Throughout infection course, progression to complicated or critical phases was seen in half and ICU admission in one-quarter of patients. Distribution of COVID-19 severity categories matched with findings of the UK Coronavirus Cancer Monitoring Project (UKKCMP). Out of 800 mainly hospitalized cancer patients, severe or critical COVID-19 was reported in 45%, whereas only 7% were admitted to ICU [12]. Former correlates with Dai et al. reporting severe COVID-19 in about 40% [5]. Admission to ICU varied between different studies, reporting the earlier mentioned 7% in a UK cancer cohort, 14% in a Canadian/Spain/American, and about 36% in a German cohort [9, 12, 14]. This apparent lack of correlation can be explained by differences in timeline of the pandemic and higher capacities of critical care beds in German hospitals [22]. Comparing age distribution of patients, who received intensive care, only a few patients older than 85 years of age were admitted. Notably, higher mortality rates were seen in patients of the same age not admitted to ICU, while transition to a palliative approach was common as well. Similar was reported by Lee et al., assuming that life-extending measures might have already been reduced in those patients [12].

In our cohort, COVID-19-related mortality was higher and survival worse than that observed in non-cancer patients. As expected, similar was seen in ICU patients. Mortality was in line with data published by Lee et al. reporting death rates of 28% for non-ICU patients and 41% for patients treated in intensive care units [12]. In accordance with previously published data, male sex, active disease, and advanced age were associated with higher mortality attributed to COVID-19 [9, 12, 14, 23].

Unlike data published in the earlier phase of the pandemic, both mortality and survival of cancer and non-cancer patients were comparable after adjustments for age, sex, and comorbidity. Thus, Mehta et al. reported significantly higher age-adjusted case fatality rates, while Dai et al. highlighted a severe disease course and worse outcome in 105 cancer patients compared with an age-matched non-cancer control group [5, 13]. Moreover, a Belgian analysis drew attention to patients with solid tumors having a higher in-hospital COVID-19 mortality compared to an age-, sex-, and comorbidity-adjusted cohort of non-cancer patients. Notably, cancer patients younger than 60 years of age were at highest risk [24].

Beyond that, a recently published retrospective analysis of cancer patients with COVID-19 treated at a Comprehensive Cancer Center in Germany performed an age-matched analysis on 39 cancer and non-cancer patients, respectively. In both groups, distribution of comorbidity and sex were well comparable. No differences in mortality and survival were observed [14].

## Conclusion

To the best of our knowledge, the present analysis is unique performing an age-, sex-, and comorbidity-adjusted comparison in a large cohort of cancer and non-cancer patients with COVID-19. Even though survival and mortality appeared to be comparable after adjustments, our results emphasize that cancer patients as a group are at higher risk due to advanced age and higher prevalence of comorbid conditions.

## Supplementary Information

ESM 1 (DOCX 30 kb)

## Data Availability

All data analyzed are included in this published article and its Supplementary Information files.
