# Supplementary material for: COVID-19 in cancer patients: clinical characteristics and outcome—an analysis of the LEOSS registry
Source: Ann Hematol. 2020 Nov 7;100(2):383–93. doi: 10.1007/s00277-020-04328-4 (PMC7648543; doi:10.1007/s00277-020-04328-4)
Supplement: Supplementary file 1 — (DOCX 30 kb) [file 277_2020_4328_MOESM1_ESM.docx]

Supplementary data

| Supplement table 1: Outcome and Course of COVID-19 in cancer patients | | |
| --- | --- | --- |
| n/N (%) | |  |
| Phase of COVID-19 at diagnosis  Uncomplicated phase  Complicated phase  Critical phase  Recovery phase  Dead | 272/433 (63)  131/433 (30.5)  22/433 (5)  3/433 (0.5)  5/433 (1) | |
| Last known patient status  Not recovered  Recovered  Dead from COVID-19  Dead from other causes | 25/431 (6)  292/431 (67.5)  97/431 (22.5)  17/431 (4) | |
| Laboratory findings at detection of SARS-CoV-2 and in critical phase | Baseline°  N=435 | Critical phase*  N=109 |
| Leukocytes  < 4,000/ µL  4000 - 11,999/ µL  ≥ 12,000/ µL | 79/363 (21.5)  241/363 (66.5)  43/363 (12) | 22/105 (21)  29/105 (27.5)  54/105 (51.5) |
| Lymphocytes  < 1500/ µL  ≥ 1,500/ µL | 228/273 (83.5)  45/273 (16.5) | 72/81 (89)  9/81 (11) |
| Neutrophils  < 2000/ µL  2000 – 8999/ µL  ≥ 9000/ µL | 49/276 (18)  199/276 (72)  28/276 (10) | 16/81 (20)  28/81 (34.5)  37/81 (45.5) |
| Platelets  <120,000/ µL  120,000 – 499,000/ µL  ≥ 500,000/ µL | 75/360 (21)  269/360 (74.5)  16/360 (4.5) | 39/94 (41.5)  47/94 (50)  8/94 (8.5) |
| Hemoglobin  < 8 g/ dL  8 – 11.9 g/ dL  ≥ 12 g/ dL | 32/361 (9)  169/361 (47)  160/361 (44) | 48/94 (51)  38/94 (40.5)  8/94 (8.5) |
| C-reactive proteine (CRP)  < 30 mg/ L  30 -120 mg/ L  > 120 mg/ L | 149/355 (42)  140/355 (39.5)  66/355 (18.5) | 18/102 (17.5)  14/102 (13.5)  70/102 (69) |
| Vital signs at detection of SARS-CoV-2 and in critical phase | Baseline°  N=435 | Critical phase*  N=109 |
| Systolic blood pressure  < 80 mmHg  80 – 139 mmHg  ≥ 140mmHg | 2/357 (0.5)  249/357 (69.5)  106/357 (30) | 30/99 (30.5)  41/99 (41.5)  28/99 (28) |
| Diastolic blood pressure  < 60 mmHg  60 – 89 mmHg  ≥ 90mmHg | 44/358 (12.5)  267/358 (74.5)  47/358 (13) | 60/81 (74)  9/81 (11)  12/81 (15) |
| Heart rate  < 60 bpm  60 – 89 bpm  ≥ 90 bpm | 10/358 (3)  220/358 (61.5)  128/358 (35.5) | 16/83 (19.5)  6/83 (7)  61/83 (73.5) |
| Oxygen saturation (SpO_2_)  < 80%  80 – 90 %  ≥ 90% | 15/338 (4.5)  38/338 (11)  285/338 (84.5) | 33/82 (40)  32/82 (39)  17/82 (21) |
| Temperature  < 38°C  ≥ 38°C | 239/361 (66)  122/361 (34) | 15/81 (18.5)  66/81 (81.5) |
| Respiratory rate  < 16/ min  16 – 21/ min  ≥ 21/ min | 52/239 (22)  119/239 (49.5)  68/239 (28.5) | 3/72 (4)  4/72 (5.5)  65/72 (90.5) |
| min= minutes, bpm= beats per minute | | |
| °data available ≤48h to the time of first positive SARS-CoV-2  *Worst during this phase of disease | | |

| Supplement table 2: Survival of COVID-19 after adjustments for age-, sex- and comorbidity | | | | |
| --- | --- | --- | --- | --- |
| Group I 36 – 55 years | | Cancer patients,  N= 49/722 | Non-cancer patients,  N= 673/722 | p-value |
|  | n/N (%)  Age  36 – 45 years  46 – 55 years | 13/49 (26.5%)  36/49 (73.5%) | 239/673 (35.5%)  434/673 (64.5%) | 0.203 |
|  | Sex  Female  Male | 19/49 (39%)  30/49 (61%) | 234/673 (35%  439/673 (65%) | 0.570 |
|  | Comorbidity  CCI w/o cancer (+/-SD) | 0.69 (+/-1.32) | 0.498 (+/-1.22) | 0.281 |
|  |  |  |  |  |
|  | Survival analysis  All patients  Median survival  Survival at 30 days; % (95%CI)  ICU patients; n/N(%)  Median survival in days  Survival at 30 days; % (95%CI) | Not reached  85 (69.5 -100.5)  14/49 (28.5)  Not reached  68.5 (53.5 – 83.5) | Not reached  89 (84.5 – 94)  186/673 (27.5)  Not reached  72 (66.5 – 77.5) | 0.259  0.326 |
|  | | | | |
| Group II  56 – 65 years | | Cancer patients,  n=72/599 | Non-cancer patients,  n= 527/599 | p-value |
|  | n/N (%)  Age  56 – 65 years | 72/72 (100) | 527/527 (100) |  |
|  | Sex  Female  Male  Comorbidity  CCI w/o cancer (+/-SD) | 32/72 (44.5)  40/72 (56.5)  0.83 (+/-1.59) | 200/527 (38)  327/527 (62)  0.75 (+/-1.42) | 0.289  0.683 |
|  | Survival analysis  All patients  Median survival  Survival at 30 days; % (95%CI)  ICU patients; n/N(%)  Median survival in days  Survival at 30 days; % (95%CI) | Not reached  85.5 (71.5 – 99.5)  24/72 (33.5)  19 (0 – 43.5)  48.5 (18.5 – 78) | 99 (25.5 – 172.5)  86.2 (81.5 – 91)  177/527 (33.5)  99.0 ( - )  67 (57 – 77) | 0.331  0.016 |
|  | | | | |
| Group III  66 – 85 years | | Cancer patients,  n= 265/1186 | Non-cancer patients,  n= 921/1186 | p-value |
|  | n/N (%)  Age  66 – 75 years  76 – 85 years | 107/265 (40.5)  158/265 (59.5) | 413/921 (45)  508/921 (55) | 0.197 |
|  | Sex  Female  Male | 101/265 (38)  164/265 (62) | 371/921 (40.5)  550/921 (59.5) | 0.525 |
|  | Comorbidity  CCI w/o cancer (+/-SD) | 1.92 (+/-2.14) | 1.76 (+/-2.03) | 0.281 |
|  | Survival analysis  All patients  Median survival  Survival at 30 days; % (95%CI)  ICU patients; n/N(%)  Median survival in days  Survival at 30 days; % (95%CI) | 51 (36.5 – 65.5)  67 (59.5 – 74.5)  78/265 (29.5)  31 (21.5 – 40.5)  54.5 (47.5 – 61) | 55.0 (–)  67.3 (63.0 – 71.6)  308/1186 (26)  24 (19.5 – 28.5)  45.5 (41.5 – 49) | 0.704  0.609 |
| Group IV  >85 years | | Cancer patients,  n= 38/249 | Non-cancer patients,  n= 211/249 | p-value |
|  | n/N (%)  Age  >85 years | 38/38 (100) | 211/211 (100) |  |
|  | Sex  Female  Male | 19/38 (50)  19/38 (50) | 124/211 (59)  87/211 (41) | 0.314 |
|  | Comorbidity  CCI w/o cancer (+/-SD) | 2.26 (+/-1.70) | 2.57 (+/-1.91) | 0.358 |
|  | Survival analysis  All patients  Median survival  Survival at 30 days; % (95%CI)  ICU patients; n/N(%)  Median survival in days  Survival at 30 days; % (95%CI) | Not reached  51 (29.5 – 72)  1/38 (2.5)  All cases are censored | 27 (15 – 39)  49.5 (40 – 49.5)  29/249 (11.5)  -  30.5 (1.5 – 59) | 0.806  0.621 |
